# Identification of a Novel Metalloproteinase and Its Role in Juvenile Development of the Tobacco Hornworm, *Manduca sexta* (Linnaeus)

**DOI:** 10.1002/jez.b.22487

**Published:** 2013-01-25

**Authors:** Smitha Vishnuvardhan, Rubina Ahsan, Kathryn Jackson, Rebecca Iwanicki, Jordan Boe, Jodie Haring, Kendra J Greenlee

**Affiliations:** Department of Biological Sciences, North Dakota State UniversityFargo, North Dakota

## Abstract

Matrix metalloproteinases (MMPs) are a class of zinc-dependent endopeptidases that are highly conserved across numerous taxa, from bacteria to humans. Recently, MMPs have been identified in several insect species and are hypothesized to function in immunity and development. In this study, we identify a putative MMP and correlate its proteolytic activity and gene and protein expression in the tracheae with developmental stage. Ms-MMP gene expression increases 10-fold during molting, which is accompanied by an increase in both protein expression and gelatinolytic activity. To directly test the hypothesis that Ms-MMP plays a critical role in juvenile development of *Manduca sexta*, we injected a broad-spectrum MMP inhibitor and recorded its effects on growth and development. Inhibition of MMPs caused a delay in juvenile development and decreased growth rates. Understanding the function of MMPs will help us better understand molting and control of body size in insects. Furthermore, elucidating functions for MMPs in lower taxa may yield critical information about the evolution of the numerous MMPs found in vertebrates. J. Exp. Zool. (Mol. Dev. Evol.) 320B:105–117, 2013. © 2013 Wiley Periodicals, Inc.

Matrix metalloproteinases (MMPs) are a class of endopeptidases belonging to the superfamily of metalloproteinases, metzincins (Bode et al., 1993). A common feature of the metzincins is the presence of the zinc-binding domain, HEXXHXXGXXH (Bode et al., 1993). The metzincins are sub-divided based on the presence of other domains into ADAMs/adamalysins, astacins, serralysins and the MMPs. MMPs can be identified based on their primary amino acid sequence, tertiary protein structure, substrate specificity, and inhibition by MMP-specific inhibitors. MMPs are named, in part, because of their ability to cleave extra-cellular matrix proteins, but their substrates include a wide range of other molecules, such as growth factors, cell adhesion molecules, chemokines, and cytokines (Sternlicht and Werb, 2001; Greenlee et al., 2007).

MMPs are thought to have evolved from a much simpler enzyme that appeared even before the divergence of vertebrates from invertebrates and are highly conserved throughout these groups (Massova et al., 1998). The first MMP discovered was a collagenase, found in 1962 in the tail of a metamorphosing tadpole larva (Gross and Lapiere, 1962). Since then, MMPs have been found in various organisms ranging from bacteria to plants to mammals, including 25 in mice and 24 in humans (Brinckerhoff and Matrisian, 2002; Mott and Werb, 2004). Activity of MMPs is often associated with tissue degradation, as seen in cancer metastasis, arthritis, or asthma. Because of this, research on therapeutic agents has focused on the inhibition of MMPs (Overall and Kleifeld, 2006). Knockout mice lacking one or more MMPs often have deleterious phenotypes when subjected to disease models (Parks et al., 2004; Greenlee et al., 2007). In contrast, even though expression of several MMPs is highly correlated with development, there is little evidence for altered developmental phenotypes in mice lacking MMP2, MMP9, and MMP14 (Vu and Werb, 2000; Kheradmand et al., 2002; Oblander et al., 2005). These various phenotypic observations are most likely due to the fact that many MMPs have been shown to have overlapping and compensatory functions. Thus, the complex milieu of MMPs in vertebrates makes understanding their function in these organisms very difficult.

A simpler system for studying MMP function can be found in insects, which have at most three MMPs per species (Knorr et al., 2009). Two MMPs have been identified in the fruit fly, *Drosophila melanogaster* (Dm1-MMP and Dm2-MMP; Llano et al., 2000; Llano et al., 2002); one in the waxmoth, *Galleria mellonella* (Gm1-MMP; Altincicek and Vilcinskas, 2008); and three in the flour beetle, *Tribolium castaneum* (Knorr et al., 2009). Both Dm-MMPs are critical for larval tracheal growth, metamorphosis, and tissue remodeling (Page-McCaw et al., 2003; Glasheen et al., 2010). Gm1-MMP and *Tribolium* MMP-1 are closely related to Dm1-MMP both in structure and function (Altincicek and Vilcinskas, 2008; Knorr et al., 2009). Inhibition of *Tribolium* MMP-1 using RNAi resulted in defects in antennae, eyes, appendages, and head. In addition, these insects were unable to complete pupation and died during metamorphosis. While both Dm1-MMP mutant larvae and MMP knockdown beetle larvae exhibited broken tracheal system trunks during juvenile development, these defects were only lethal in *Drosophila* (Altincicek and Vilcinskas, 2008; Glasheen et al., 2010).

Because of our interest in MMPs due to their importance in disease processes and development, and our specific interest in growth during juvenile development, we sought to identify a putative MMP in the model organism, *Manduca sexta*, the tobacco hornworm. In this study, we correlate proteolytic activity and gene and protein expression in the tracheae with developmental stage. To directly test the hypothesis that Ms-MMP plays a critical role in juvenile development of *M. sexta*, we injected a broad-spectrum MMP inhibitor and recorded its effects on growth and development.

## MATERIALS AND METHODS

### Insect Rearing

*M. sexta* (Linnaeus) eggs were obtained from Carolina Biological Supply Company (Burlington, NC, USA). Larvae were reared as previously described with ad libitum access to a wheat germ diet (Greenlee and Harrison, 2005). Larvae were maintained under a 16L:8D photoperiod cycle at 25°C. Larval age was determined visually by monitoring the presence of the head capsule which indicated the animal was preparing to molt. We used fourth and 5th instar larvae for all of the experiments due to their large size.

### Cloning and Sequencing of Ms-MMP

Total RNA was extracted from fat bodies of pupal *M. sexta* or from tracheae of larvae using TRIzol® reagent (Invitrogen, Carlsbad, CA, USA) as per the manufacturer's protocol, except that samples were centrifuged at room temperature. Isolated RNA was resuspended in RNase-free deionized water at a concentration of 1 µg/µL. Complementary DNA was prepared from 1 µg of RNA using reverse transcription with Masterscript RT-enzyme and random primers (5-Prime, Gaithersburg, MD, USA). Degenerative primers (Integrated DNA Technologies, Coralville, IA, USA) designed for *G. mellonella* (Altincicek and Vilcinskas, 2008) were used to amplify putative Ms-MMP sequences from 1 µL of the cDNA (Table 1), using gradient PCR for 40 cycles of amplification (0.5 min at 93°C, 2 min at 43–63°C, and 3 min at 68°C). PCR products were visualized with agarose gel electrophoresis, cloned into the TOPO vector (Invitrogen), and transformed into one-shot chemically competent *Escherichia coli*, according to the manufacturer's protocol. Purified plasmids (75 fmol) were sequenced using the CEQ 2000 Dye Terminator Cycle Sequencing with Quick Start Kit (Beckman Coulter, Fullerton, CA, USA), according to the manufacturer's protocol.

**Table 1:** Primers and probes used in this study

Primer or probe name	Sequence 5′–3′	
dMMP/fwd	GAYGCNCAYTTYGAYGAYG	Degenerative primers: K = G or T; R = A or G; Y = C or T; N = A or G or C or T
dMMP/rev	GRTCRAANCKCCARAAYTT	
N-2RC	AGAATTTCGAGCCCTTGTAGAAATAGATCTTG	5′ RACE
1NRS	GCCGAGCGGACGTCGCTGTGCGA	
2NRS	TCGGCGCAGAGCGCGGGGTCTTCC	
3NRS	GCCGCCACTCCGTCCTCGGTTA	
seq1NRS	GAGCGGACGTCGCTGTG	
seq2NRS	CAGAGCGCGGGGTCTTC	
seq3NRS	GCCACTCCGTCCTCGGTT	
1FS	GCACAGCGACGTCCGCTCGGCCCTCAT	3′ RACE
1FS	GCACAGCGACGTCCGCTCGGCCCTCAT	
2NFS	CGCGCTCTGCGCCGATCCCAGA	
3NFS	GACGGAGTGGCGGCCGGCTACC	
1FC	TATTTCTACAAGGGCTCGAAATTCTGGCGC	PCR for tissue distribution
MMP/rev	AGAATTTCGAGCCCTTGTAGAAATAGATCTTG	
MMP/RT/fwd	ATCCCAGAATCGACACCATCT	Real-time PCR for Ms-MMP
MMP/RT/rev	GCTGCCTTTGAACACAAATG	
MMP/probe	CGCTGACGGCTCC	
rpS3/RT/fwd	CCGCATCCGCGAGTTG	Real-time PCR for rpS3
rpS3/RT/rev	CGGACTGTTCCGGGATGTT	
rpS3/probe	CGTGCAGAAGCGGTT	

To validate our sequencing results, we designed primers from the obtained sequence that generated a 356 bp fragment (Table 1). Resulting PCR products from fat body (FB), hemocytes (HC), and tracheae (TR) were subjected to gel purification. PCR products were cloned into PGEMTeasy vector (Promega Corporation, Madison, WI, USA) and sequenced as above using universal M13 forward primer.

### RACE PCR

DNA contamination was removed from total RNA using DNase I, Amplification Grade (Invitrogen). RNA quality was determined on a denatured 1.2% agarose gel. Total RNA (1–2 µg) was used to synthesize 5′ and 3′ RACE cDNA each using SMARTER™ RACE cDNA amplification kit (Clontech, Mountain View, CA, USA), according to manufacturer's instructions. All the PCR reactions were carried out using Advantage 2 polymerase mix and Advantage Ultrapure PCR deoxynucleotide mix (Clontech), according to manufacturer's instructions.

To extend the 5′ and 3′ regions of Ms-MMP, we ran touch-down PCR, using either 5′ or 3′ RACE cDNA, gene-specific primers (Table 1), and the universal primer A mix (UPM; Clontech). The resulting PCR product was diluted 50-fold with tricine–EDTA buffer and used as a template for nested PCR. For 5′ RACE, three reverse primers were designed upstream at 120, 315, and 516 bp respectively from 3′ available DNA sequence of Ms-MMP, and PCR reactions (50 µL) were carried out using the gene-specific primers 3NRS, 2NRS, and 1NRS (10 µM, Table 1) and 2 µL of UPM. For 3′ RACE, three forward primers were designed downstream at 269, 456, and 597 bp, respectively, from 5′ available DNA sequence of Ms-MMP. Three PCR reactions were carried out using diluted touch-down 3′ RACE PCR as template; gene-specific primers 2NFS, 1NFS, 1FC (Table 1); and the UPM. Thermocycler parameters were as follows: 94°C for 1 min, followed by 35 cycles of 94°C for 30 sec, 68°C for 30 sec, and 72°C for 3 min, with a final extension at 72°C for 5 min. PCR products were separated on an agarose gel, and the resulting bands were gel-purified using Wizard SV gel and PCR clean-up system (Promega Corporation) and sequenced as described above. The PCR product was cloned into pGEM^T^_-_easy vector system II (Promega Corporation) and transformed into JM109 chemically competent cells, according to manufacturer instructions (Promega Corporation). Recombinant DNA was isolated using QIAprep spin miniprep kit (Qiagen, Valencia, CA, USA) according to manufacturer instructions. The presence of the Ms-MMP insert in plasmid was confirmed by EcoR1 restriction digestion (Promega Corporation) for 3–4 hr at 37°C. Recombinant DNA containing fragments of the expected size were sent for sequencing at the Core Facility, Tufts University Boston, MA.

### Full-Length Ms-MMP Assembly

Sequences obtained from Tufts were compared to those of *Bombyx mori* MMP and other insects. Homologous sequences were aligned to the partial Ms-MMP sequence and assembled using ClustalW2 and Geneious Pro software (Geneious version 4.0.2 created by Biomatters. Available from http://www.geneious.com) after removing the vector sequence. Mismatched nucleotides within the previously obtained partial sequence were replaced with the consensus 5′ and 3′ nucleotide sequence to yield full length Ms-MMP cDNA. The open reading frame was identified using the Sequence Manipulation Suite ORF Finder (Stothard, 2000).

### Sequence Alignment, Phylogram, and Structural Analysis

Sequences from several MMPs and metzincin family members (see Table 2 for accession numbers) were aligned using CLUSTALX version 2.0.12 with the Gonnet series protein weight matrix. A neighbor joining tree was constructed from the CLUSTAL alignment, using Geneious Pro software (Geneious version 4.0.2 created by Biomatters. Available from http://www.geneious.com). The tree was rooted using serralysin from *Serratia proteamaculans* as the outgroup, and bootstrapping was performed with 1,000 iterations. The tree presented here was constructed from the 75% majority rule of all sampled trees (Hall, 2001). To determine if our MMP is more closely related to insect MMP1, MMP2 or MMP3, we again used CLUSTALX to align the sequences. An unrooted, neighbor joining tree was constructed from the alignment. The deduced amino acid sequence was analyzed using Simple Modular Architecture Research Tool (SMART; Schultz et al., 1998) to determine conserved protein domain structures.

**Table 2:** Accession numbers, species names, and protease names for aligned sequences used in the phylogenetic analyses

Accession number	Species	Protease
ACS52560.1	*Anopheles gambiae*	MMP1
XP_320653.4	*Anopheles gambiae*	MMP2
XP_554330.4	*Anopheles gambiae*	MMP3
XP_001861713.1	*Culex quinquefasciatus*	MMP1
NP_001036935	*Bombyx mori*	Adamalysin
NP_001116499	*Bombyx mori*	MMP1 (isoform 1)
NP_001116500.1	*Bombyx mori*	MMP1 (isoform 2)
NP_001189002.1	*Drosophila melanogaster*	MMP1 (Dm1-MMP)
NP_995788	*Drosophila melanogaster*	MMP2 (Dm2-MMP)
CAL29436.1	*Galleria mellonella*	Collagenase 1
CAL29437.2	*Galleria mellonella*	Collagenase 1.2
EFN76651.1	*Harpegnathos saltator*	MMP14
AAH02576	*Homo sapiens*	MMP2 (gelatinase A)
EAW75776	*Homo sapiens*	MMP9 (gelatinase B)
NP_004986.1	*Homo sapiens*	MMP14 (MT1-MMP)
XP_003704614.1	*Megachile rotundata*	MMP1
XP_003706265.1	*Megachile rotundata*	MMP2
NP_001171780.1	*Saccoglossus kowalevski*	MMP19-like
YP_001478616.1	*Serratia proteamaculans*	Serralysin
ACG69475.1	*Spodoptera frugiperda*	Astacin
NP_001157647.1	*Tribolium castaneum*	MMP1
XP_969495.1	*Tribolium castaneum*	MMP2
XP_972146.1	*Tribolium castaneum*	MMP3

### Collection of Tracheal Tissue

Caterpillars were collected during each day of development during the 4th and 5th instars, anesthetized on ice for 10–15 min, and surface-sterilized with 75% ethanol. All dissection tools used for sample collection were sterilized with 75% ethanol. An incision was made on the dorsal surface of the caterpillar along the heart, and the larva was opened carefully. The gut was removed and the body was washed with cold saline buffer (4 mM NaCl, 40 mM KCl, 18 mM MgCl_2_·6H_2_O, 3 mM CaCl_2_, pH 6.5) with a few crystals of phenylthiourea (PTU). The fat body was carefully removed to reveal intact tracheae. Using forceps, tracheae were collected into microcentrifuge tubes and macerated with polystyrene pestles in 200 µL of saline buffer without PTU. Samples were centrifuged at 12,000*g* for 5 min at 4°C and the supernatant transferred to new microcentrifuge tubes.

### PCR and Real-Time Semi-Quantitative-PCR (q-PCR)

Total RNA from tracheal samples from four caterpillars each day was prepared using TRIzol® reagent (Invitrogen) according to the manufacturer's protocol and stored at −80°C. Two micrograms of total RNA was used to make cDNA in a reverse transcription reaction using Oligo(dT)_15_ primers (GoScript Reverse Transcription System, Promega Corporation or iScript cDNA Synthesis Kit, Bio-Rad Laboratories, Hercules, CA, USA). Real-time quantitative-PCR was performed with TaqMan® Universal 2× Master Mix (Applied Biosystems, Carlsbad, CA, USA) on an ABI 7500 Fast Real-Time PCR system (Applied Biosystems) using standard cycling conditions. Ms-MMP gene expression was normalized to ribosomal protein subunit 3 (rpS3) as previously described (Jiang et al., 1996). Fold changes in Ms-MMP expression were generated using the comparative Ct method of PCR data analysis with the normalizer sample set to a sample from the youngest group (4th instar, day 3) with three technical replicates. Validation experiments were performed to ensure that efficiencies of Ms-MMP and rpS3 amplification remained consistent over a wide range of template concentrations. Controls included water and RNA only samples as templates. PCR primer (Sigma-Genosys, The Woodlands, TX) and probe sequences (Applied Biosystems) used in these experiments are listed in Table 1.

### Protein Precipitation and Estimation of Protein Concentration

Proteins were precipitated from all samples using four volumes of methanol at room temperature for 15 min. To recover precipitated proteins, samples were centrifuged at 12,000*g* for 10 min and the supernatant was discarded. The resulting pellet was resuspended in 200 µL of saline buffer without PTU. Protein concentration was estimated using a Bradford assay as per the manufacturer's instructions (Quick Start Bradford Protein Assay, Bio-Rad Laboratories). Samples and bovine serum albumin standards were run in duplicate.

### Protein Expression

**Figure 1:**
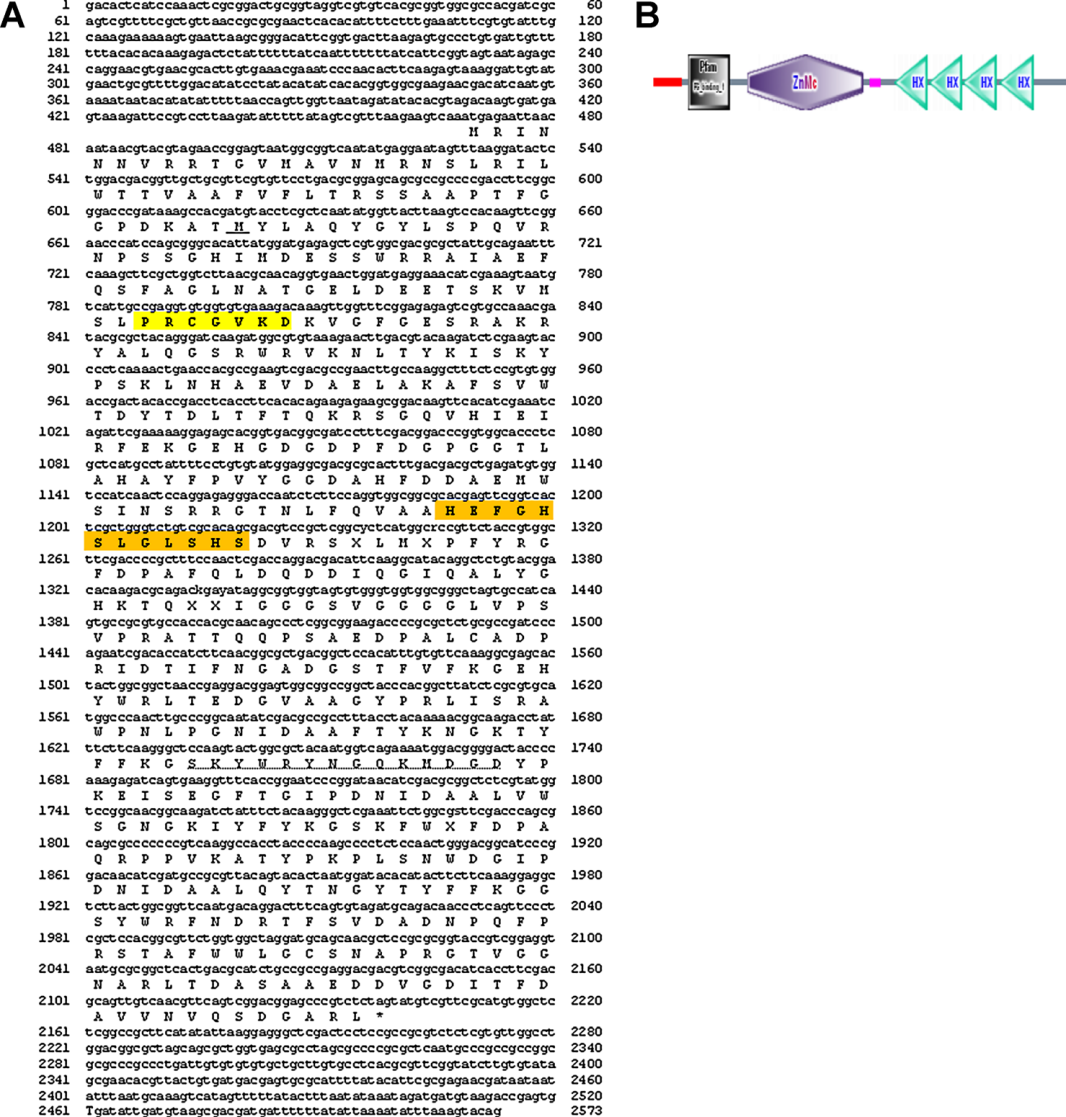
**A**: Nucleotide sequence and the deduced amino acid sequence of Ms-MMP. The underlined M residue indicates the putative translation start site. The amino acid sequence highlighted in yellow indicates the cysteine switch, while the orange highlighting indicates the conserved catalytic domain. The amino acid sequence underlined with a dotted line indicates the peptide used for antibody synthesis. **B**: Structural analysis of Ms-MMP shows the signal peptide (red; position 1–39) overlapping the transmembrane domain (position 21–43), a peptidoglycan binding domain (Pfam; position 47–104), the catalytic domain (ZnMc; position 128–285), and four hemopexin repeats (HX; positions 326–370, 372–416, 418–465, and 467–514).

Protein expression profiles of Ms-MMP in the 4th and 5th instar larvae were studied using Western blotting. Protein samples (10 or 20 µg each) were mixed with Laemmli sample buffer with 1% β-mercaptoethanol (Bio-Rad Laboratories) in a 2:1 ratio, denatured at 95°C for 5 min, and loaded onto a polyacrylamide gel (12% resolving and 5% stacking). Gels were electrophoresed at 100 V for 1.5–2 hr at room temperature. Proteins were transferred to a 0.45 µm nitrocellulose membrane (Whatman GmbH, Dassel, Germany), and the membrane was blocked overnight at 4°C in 25 mL of 5% dry milk in 1× Tris-buffered saline with 0.5% Tween-20 (TBST; 0.1 M Tris, 1.5 M NaCl, pH 8). A rabbit, polyclonal anti-Ms-MMP was custom-synthesized using the peptide CSKYWRYNGQKMDGD (Fig. 1; ProSci Incorporated, Poway, CA, USA). The purified antibody was diluted 1:500 and used as the primary antibody. Horseradish peroxidase-conjugated goat-anti-rabbit-IgG antibody (Pierce Protein Research Products, Rockford, IL, USA) was diluted 1:100,000 and used as the secondary antibody. Proteins were visualized using chemiluminescence (Super Signal West Femto, Thermo Scientific, Rockford, IL, USA), and the signal was detected for 1 and 3 min (ChemiImager™, Alpha Innotech, Santa Clara, CA, USA).

### Zymography

Gelatinolytic activity of MMPs in the tracheal samples was analyzed using zymography. Substrate gels (10% acrylamide with 0.3% gelatin) were loaded with samples containing 5 µg protein in a 1:1.5 ratio with zymogram sample buffer (Bio-Rad Laboratories) under native conditions. After electrophoresis, the gels were washed in renaturation buffer (2.5% Triton X-100) for 30 min at room temperature and incubated in developing buffer (50 mM Tris, 5 mM CaCl_2_, 0.02% sodium azide; pH 8) at 37°C overnight. Then, the gels were stained using 0.5% coomassie brilliant blue (R-250) and destained in 40% methanol and 10% acetic acid.

### Preparation of GM6001

A stock solution of 100 µM GM6001 (Core Synthesis Facility, NDSU, Fargo, ND, USA or Millipore, Billerica, MA, USA) was prepared by dissolving 5 mg of GM6001 in 128.68 µL of dimethyl sulfoxide (DMSO) and stored at −20°C. A working solution (10 µM GM6001) was prepared by diluting the stock with 1× PBS, resulting in a final concentration of 0.001% DMSO.

### Injection of GM6001

To test the effect of MMP inhibition on the molting process, caterpillars from 4th instar larvae of similar body masses were injected with 10 µL of inhibitor (10 µM GM6001 in PBS with 0.001% DMSO) or vehicle (PBS with 0.001% DMSO). The growth and timing of molting in inhibitor- and vehicle-treated caterpillars were compared to that of caterpillars that received no injections. Injections began on the third day of the 4th instar and continued daily until the dorsal heart was observed in the 5th instar. Caterpillars were weighed daily and monitored for any visual changes in the control versus treatment groups. All of the caterpillars survived the experiment and appeared healthy on visual inspection.

### Data Analysis

Statistical analyses were performed using IBM SPSS versions 19 and 20. Differences between the treatment groups were detected by Analysis of Variance (ANOVA). Effects of MMP inhibitor on daily mass and daily growth rates were tested using repeated measures ANOVA with time as the within subjects repeated factor and treatment as the between subjects factor. Development time was assessed using a Kaplan–Meier time-to-event analysis. A probability value of 0.05 was used to indicate the significance, unless otherwise indicated. Data are expressed as mean ± standard error of the mean throughout.

## RESULTS

### Identification of Ms-MMP

**Figure 2:**
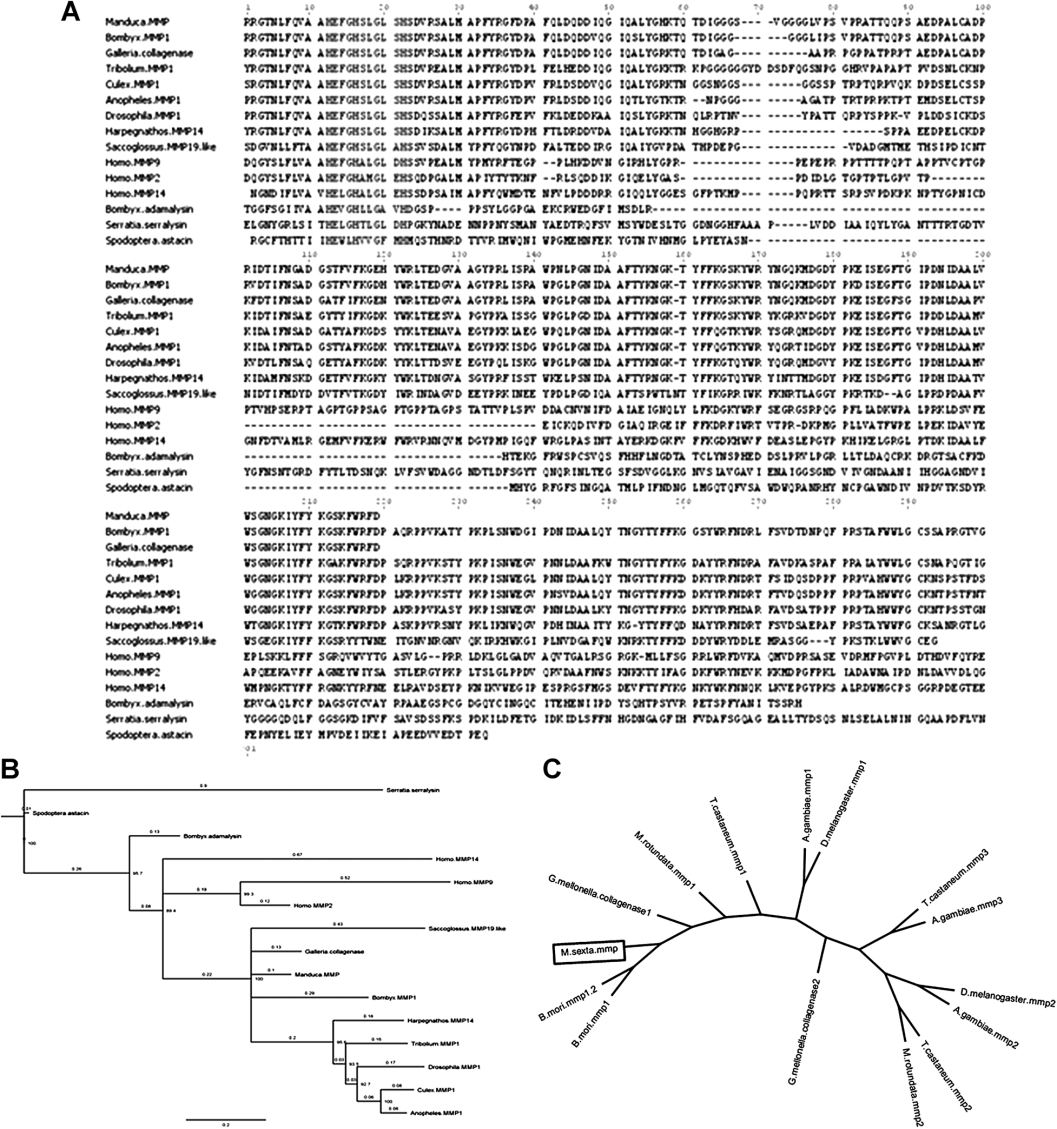
**A**: Amino acid sequence alignment used to generate the phylogram. Shaded box denotes the conserved zinc-binding catalytic domain. **B**: Phylogenetic relationships between metzincin superfamily members, including several MMPs, astacins, serralysin, and adamalysin. Numbers above branches indicate substitutions per site, while numbers to the right of nodes indicate the consensus percent. **C**: Phylogenetic relationships between insect MMPs.

Using degenerative primers, a 643 bp cDNA fragment was obtained and sequenced. From that sequence, using nested RACE PCR, we isolated the full-length cDNA of Ms-MMP (Fig. 1A; GenBank: JN415760.1). The corresponding 2,513 bp mRNA has an ORF 2 in reading frame 1 extended from base 468 to base 2,197 (Fig. 1A). The most likely translation start site is the 4th Met residue (underlined in Fig. 1A), because the sequence immediately upstream of that residue is CAA, which is identical to the *Drosophila* translation start site consensus sequence of (C/A)AA(A/C). The identified ORF encoded 556 deduced amino acid residues, with a predicted molecular mass of 61.5 kDa. The deduced amino acid sequence was searched using SMART and contains several key MMP characteristics (Fig. 1B). Our sequence consists of an 87 residue (40–127) N-terminal pro-domain followed by the 157 residue (128–285) catalytic domain and four C-terminal hemopexin-like domains (Fig. 1B; amino acid positions 326–370, 372–416, 418–465, and 467–514). A conserved domain database search also identified TIMP and metal binding sites within the catalytic domain. Ms-MMP has a conserved cysteine switch motif PRCGVXD in the pro-domain, which is characteristic of most MMPs (Fig. 1A; position 106–112). The pro-domain also contains a furin consensus sequence RXKR (position 119), which mediates intracellular activation. The catalytic domain contains the conserved motif HEXXHXXGXXHS, where X is a variable amino acid. The three His residues, zinc binding sites, the Ser residue, and a Met reside seven amino acids past the serine distinguish MMPs from other metalloproteinases. Finally, there is a hinge region of 35 residues (position 284–319) inserted between the catalytic and hemopexin-like domains. BLAST analysis in GenBank™ of Ms-MMP yielded matches with numerous other MMPs, and alignment of our sequence with other MMPs identified from BLAST searching revealed similarity in the zinc-binding domain as well as other regions (Fig. 2A). Phylogenetic analysis showed that Ms-MMP grouped with the other MMPs and not with the other proteases that share the zinc-binding domain, such as astacin, adamalysin, or serralysin (Fig. 2B). The evidence strongly suggests that the sequence we identified is a MMP. In addition, Ms-MMP clusters with MMP1 from other insects, rather than MMP2 or MMP3 (Fig. 2C).

**Figure 3:**
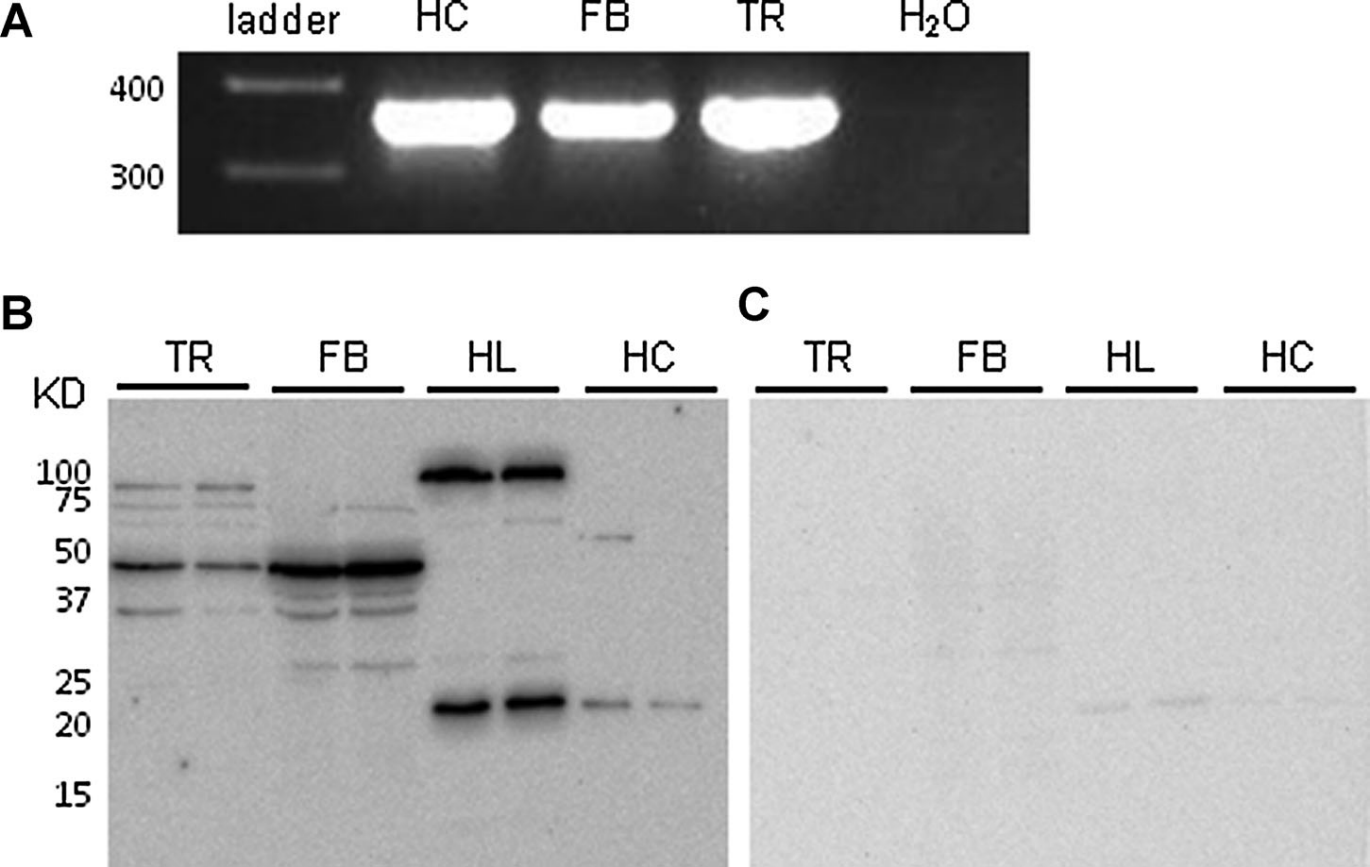
Tissue distribution of Ms-MMP in 5th instar larvae. **A**: Ms-MMP was amplified from cDNA from hemocytes (HC), fat body (FB) and tracheae (TR). **B**: Anti-Ms-MMP detected Ms-MMP in TR, FB, hemolymph (HL), and HC. **C**: Pre-incubation of the antibody with the Ms-MMP peptide abolished most signal from the membrane.

Using gene-specific primers designed from the Ms-MMP sequence (Table 1), we detected Ms-MMP gene expression in tracheae, fat body and hemocytes (Fig. 3A). Sequence analysis of these PCR products confirmed our initial sequencing results. Western blot analysis of tissue samples revealed most Ms-MMP protein expression in fat body and tracheae (Fig. 3B) as a 46 kDa band, with a smaller band at 35 kDa. In hemolymph, Ms-MMP was detected at 100 and 22 kDa, and hemocytes showed a single band at 22 kDa (Fig. 3B). The bands are specific to Ms-MMP, since subsequent probing of the blot with antibody that was pre-incubated with the peptide-antigen complex resulted in the disappearance of the bands (Fig. 3C).

### MMP Expression Varies Throughout Development

**Figure 4:**
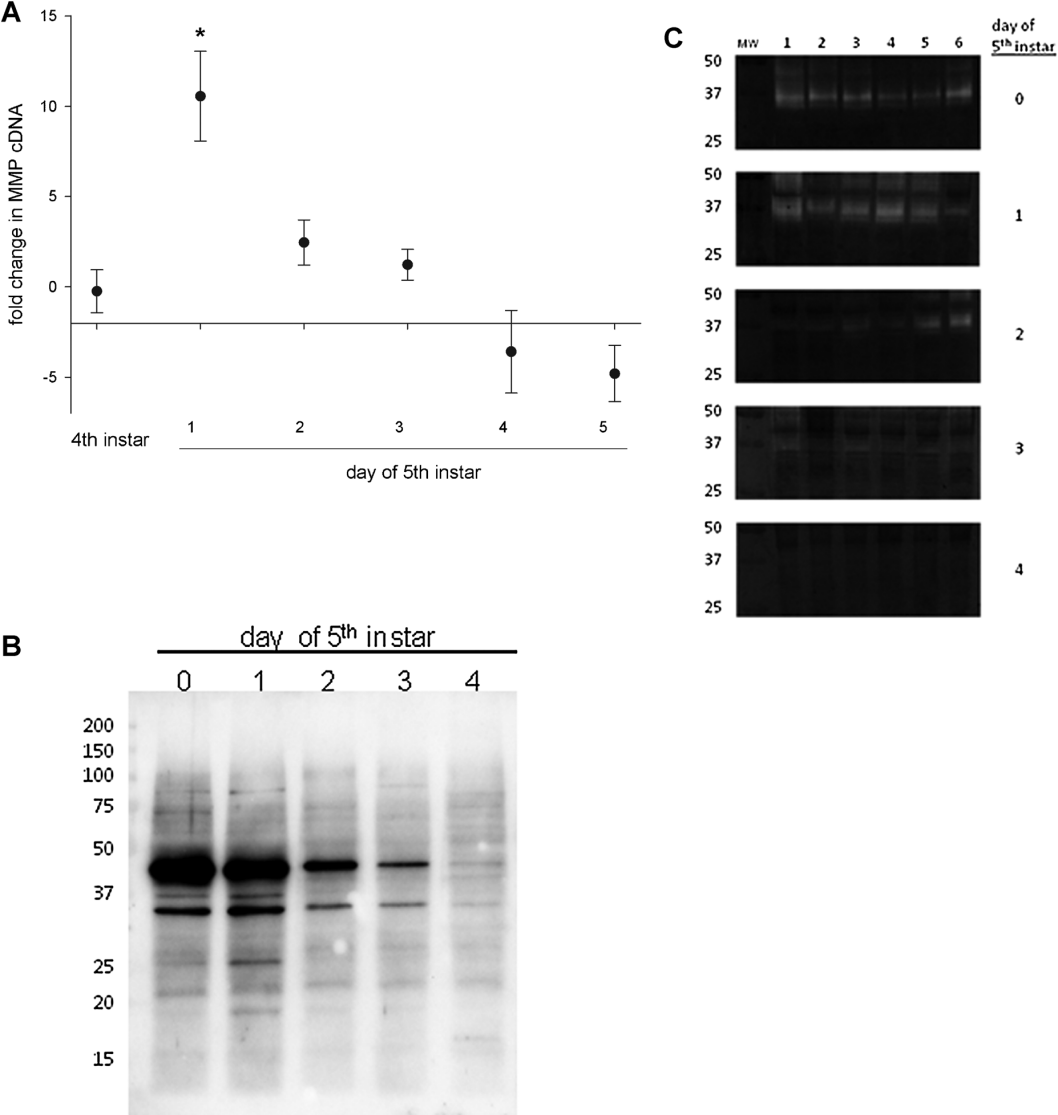
Expression of Ms-MMP varies throughout development. **A**: Ms-MMP gene expression in tracheae peaked on the first day of the 5th instar (*n* = 4 caterpillars per day). Asterisk indicates that day 0 is significantly higher than other days (*P* < 0.02). **B**: Western blot of tracheae using rabbit anti-Ms-MMP (numbers to left are the molecular masses in kDa). Each lane represents tracheae from one caterpillar. **C**: Zymograms of tracheae collected daily during the 5th instar show gelatinolytic activity early in the instar (clear bands). Each panel shows tracheal samples from six individual caterpillars. Numbers to left are the molecular masses in kDa. Experiments were repeated at least three times.

In the tracheae, Ms-MMP gene expression varied by day of development (Fig. 4A; *F*_5,23_ = 10.27, *P* < 0.001), peaked on day 0 of the 5th instar and decreased until samples were no longer collected on day 4 (Bonferroni corrected post hoc tests, *P* < 0.02). Ms-MMP protein expression followed the same pattern, where expression was highest on day 0 of the 5th instar and gradually decreased toward the end of the 5th instar (Fig. 4B). Western blotting again revealed two protein bands in 5th instar tracheal samples, one at 35 kDa and another at 46 kDa (Fig. 4B). Since some MMPs are known to be gelatinolytic, we used gelatin zymography to detect enzymatic activity. Similar to the results observed in the Western blots, the zymograms of tracheal proteins from 5th instar larvae showed high proteolytic activity during the beginning of the instar, which decreased as the instar progressed (Fig. 4C). A prominent proteolytic band corresponding to molecular weight ∼35 kDa was detected, similar to the molecular weight of the lower band observed in the Western blot (Fig. 4B).

### In Vivo Effects of MMP Inhibition on Larval Growth

**Figure 5:**
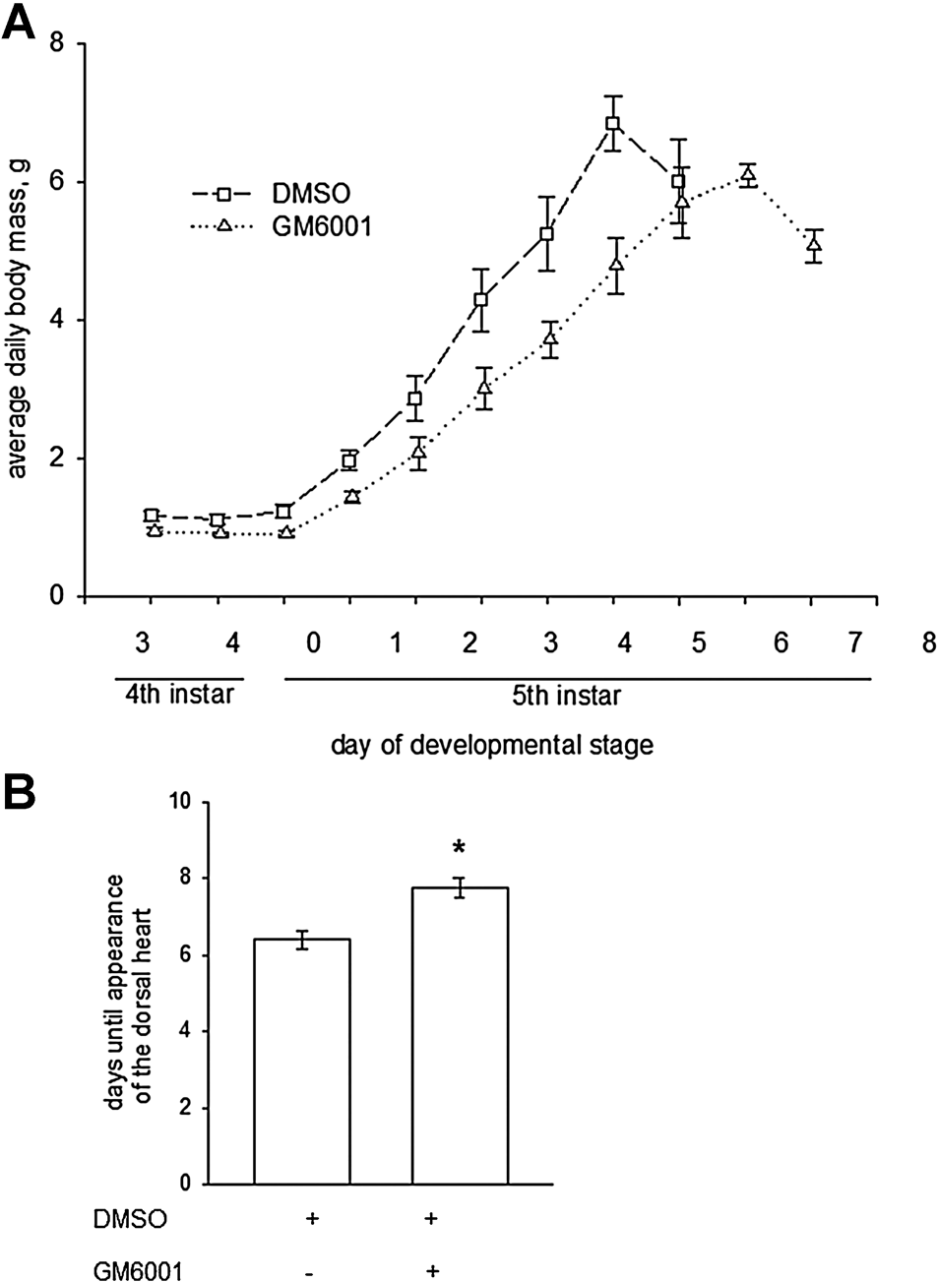
Effect of MMP inhibition on the growth of caterpillars. **A**: Caterpillars injected with GM6001 (triangles) weighed significantly less than control caterpillars (circles, *n* = 5 per group) by day 4 of the 5th instar. **B**: Juvenile development was significantly delayed, as indicated by the delayed appearance of the dorsal heart in the MMP-inhibited larvae. Asterisk indicates significant differences (*P* < 0.01).

Daily injections of the inhibitor significantly affected the growth of the caterpillars over time (Fig. 5A). Caterpillar growth varied differently depending on the treatment, with inhibitor-treated caterpillars having lower daily growth rates than caterpillars that were treated with vehicle (Fig. 5A; repeated measures ANOVA; time × treatment interaction, *F*_7, 49_ = 4.95, *P* < 0.001). Treatment with the inhibitor also affected the timing of development, as marked by the appearance of the dorsal vessel (Fig. 5B; Kaplan–Meier log rank test; χ^2^ = 5.7, *P* < 0.02). Inhibitor-treated caterpillars were delayed nearly 1.5 days compared to vehicle-treated caterpillars (Fig. 5B). However, both vehicle and inhibitor-treated caterpillars eventually reached the same mass (5.6 ± 0.25 g) when the dorsal vessel appeared.

## DISCUSSION

In this study, we identify a novel MMP in the caterpillar, *M. sexta*, that is involved in juvenile development, slowing 5th instar growth and delaying the larval–pupal transition. Ms-MMP gene and protein expression both increase at the time of molting and decrease throughout the instar, suggesting that it may be important for molting itself or for tissue remodeling that occurs immediately after molting. In vivo inhibition of Ms-MMP resulted in decreased growth rates and delayed molting, suggesting that there may be dual roles for this protease.

To verify that the protein we identified was a MMP, we used phylogenetic analyses. Because the zinc-binding motif is also present in other zinc-dependent endopeptidases, including the adamalysins, the astacins, and the serralysins (Bode et al., 1993), these proteases were included in the phylogenetic analysis. Ms-MMP grouped with the other insect MMP1s, providing additional support to the hypothesis that the protease we identified is a MMP. Together, the sequence alignment and the phylogenetic analysis provide strong evidence that the protease we identified is a MMP (Figs. 1 and 2).

While our search only identified one MMP, most insects studied have two or three MMPs (Llano et al., 2002; Knorr et al., 2009). A BLAST search on NCBI turns up an unrelated zinc-dependent protease, but no other matches in *M. sexta*. However, using the conserved catalytic domain of Ms-MMP, a BLAST search of the current draft of the *M. sexta* genome (Agricultural Pest Genomics Resource Database: http://www.agripestbase.org) identified three possible similar sequences, supporting the hypothesis that other MMPs exist. Since Ms-MMP clusters with MMP1 from other insects (Fig. 2C), it is likely that other MMPs will be homologous with insect MMP2 or MMP3.

MMPs are expressed as zymogens (Woessner and Nagase, 2000), and both the pro- and active forms can be detected using Western blotting or zymography. The presence of multiple bands in our Western blots (Figs. 3B and 4B) indicates that this is likely also the case for Ms-MMP. We are confident that the polyclonal antibodies detected Ms-MMP proteins because pre-incubation of our custom synthesized antibodies with either the peptide-antigen (Fig. 3C) or mouse-MMP2 (data not shown) resulted in the disappearance of most bands. The size of the full length Ms-MMP protein is comparable to that found in *D. melanogaster*, 60.3 kDa (Llano et al., 2000). As in *D. melanogaster*, we detected multiple sizes of Ms-MMP (Figs. 3B and 4B). For example, active, recombinant Dm1-MMP expressed in *E. coli* was identified on Western blot as a 19 kDa band, while larval extracts exhibit two bands of Dm1-MMP (61 and 49 kDa). From the deduced amino acid sequence, one can predict the size of the active band by calculating the MW of the sequences downstream of the cysteine switch (starting at residue 40, Fig. 1). By this method, activated Ms-MMP is predicted to be 49.8 kDa. However, post-translational processing, including phosphorylation and proteolytic cleavage, alternative splicing, and the formation of dimers are common among MMPs and may result in multiple bands (Woessner and Nagase, 2000; Greenlee et al., 2007; Cauwe and Opdenakker, 2010). Alternatively, the extra bands detected in Figures 3B and 4C could be other MMPs, since the peptide used to generate the antibody is from the highly conserved region of the protein. Clearly, more work needs to be done to understand the functional significance of the tissue-specific Ms-MMP expression patterns.

To date, the role of MMPs in insect development has been investigated in only a few studies, including the present study (Page-McCaw et al., 2003; Knorr et al., 2009; Glasheen et al., 2010). Two studies showed that lack of MMP1, either by mutation (*D. melanogaster*) or RNAi (*T. castaneum*), caused defects in tracheal respiratory structures, which were lethal in *D. melanogaster*. Dm1-MMP is required for elongation and growth of tracheae, as well as degradation of cuticle for proper ecdysis (Glasheen et al., 2010). In addition, *T. castaneum* and *D. melanogaster* were unable to successfully pupate, suggesting that there are multiple roles for MMPs in insect development (Knorr et al., 2009; Glasheen et al., 2010). The mechanism of MMP action in insects is largely unknown, but MMPs in mammals have been shown to cleave chemotactic factors (Greenlee et al., 2006) and cytokines (reviewed in Greenlee et al., 2007), as well as serine proteases (Brown et al., 1995) and their inhibitors (Liu et al., 2000), all of which may be important for tissue remodeling or degradation. Indeed, serine proteases are a major component of molting fluid (Katzenellenbogen and Kafatos, '^70^, '^71^; Bade and Shoukima, 1974; Bade and Stinson, 1978; Samuels et al., '93a,b; Samuels and Reynolds, 2000).

Inhibition of Ms-MMP in our system resulted in decreased growth and delayed appearance of the dorsal vein. While the underlying cause of the delay has not yet been investigated, it could have resulted from tracheal defects, as was found in *D. melanogaster* (Page-McCaw et al., 2003). Tracheal defects could result in decreased oxygen availability and cellular hypoxia, which has been shown to slow development in other insects (Loudon, 1988; Greenberg and Ar, 1996; Klok et al., 2009). Alternatively, the decrease in growth could be due to dysfunction or dysregulation of metabolic enzymes that are substrates of MMPs. MMPs may cleave many intracellular proteins, including enzymes required for metabolism and oxidation of reactive oxygen species (reviewed in Cauwe and Opdenakker, 2010). Inhibition of Ms-MMP at the transcriptional level would yield more clear results, since ensuring complete inhibition of enzymatic activity is difficult with injection of chemical inhibitors.

The increased expression of Ms-MMP near the 4th to 5th instar molt hints at its role in the process of molting. Previous use of the MMP inhibitor, 1,10-phenanthroline in *M. sexta* resulted in an 83% reduction of proteolytic activity of the molting fluid from 5th instar larvae and 98% of proteolytic activity from molting fluid of pupae that were nearing adult emergence (Samuels et al., 1993a), suggesting that one or more MMPs may be involved in molting, either directly or indirectly. The process of molting includes both the separation and shedding of the old cuticle, apolysis and ecdysis, respectively (Chapman, 1998). These processes require several enzymes, many of which have been identified from the fluid filling the space between the old and new cuticle, called molting fluid. These enzymes include chitinases, phosphatases, and phenoloxidases (Reynolds and Samuels, 1996), which could be activated or inactivated by MMPs. Furthermore, two trypsin-like serine proteases have been identified from the molting fluid of *B. mori* (Katzenellenbogen and Kafatos, 1971), and serine proteases are commonly substrates of MMPs (Overall and Blobel, 2007).

Despite the strong evidence for a potential role for Ms-MMP in larval–larval molting, our in vivo inhibition experiment did not prevent the 4th to 5th instar molt. Inhibitor injections began on the third day of the 4th instar with the goal of inhibiting the molt to the 5th instar. However, caterpillars molted synchronously. Because the 4th instar is short (the 4th instar larvae start to molt on the 4th day), injections from the first day of the 4th instar may help to elicit a stronger effect of the inhibitor on molting.

Although we were unable to prevent larval–larval molting, in vivo inhibition of Ms-MMP resulted in a decrease in growth (Fig. 5A). The decreased growth rates of inhibitor-treated caterpillars manifested as a delay in the events leading up to pupation, in which the dorsal heart appeared a day and a half after those of control caterpillars (Fig. 5B). Similar results were obtained in the flour beetle *T. castaneum*, where the larval–pupal transition was inhibited when MMP-1 was knocked down using RNAi (Knorr et al., 2009) and in *M. sexta* fed 1,10-phenanthroline, which had delayed and arrested development (Bade and Shoukima, 1974). These results yield several hypotheses about the role of MMP in developing insects. One hypothesis is that Ms-MMP may regulate PTTH, which initiates gut purging along with the appearance of dorsal heart (Nijhout and Williams, 1974). Alternatively, Ms-MMP may regulate one or more proteases involved in tissue degradation, including clearance of tissue surrounding the dorsal blood vessel (Nijhout and Williams, 1974). Since we observed a general decrease in growth rate, but inhibited caterpillars eventually reached the same mass at pupation, Ms-MMP may activate or inactivate intracellular enzymes, such as those involved in carbohydrate metabolism or protein synthesis (Cauwe and Opdenakker, 2010). Lastly, MMPs are known to be important in the innate immune response in mammals (Li et al., 2004; Hong et al., 2011) and in insects (Altincicek and Vilcinskas, 2008; Knorr et al., 2009), and it is possible that the decreased growth occurred because the inhibitor-treated caterpillars had sub-lethal infections and exhibited a trade-off between immunity and growth.

While MMP research in mammals is extensive, research on MMPs in invertebrates is just beginning. MMPs are important proteases, functioning in processes as varied as development, wound healing, and immunity, and yet many of their functions and substrates are still unknown. Our data along with the handful of other studies on insect MMPs show an intriguing pattern of MMP involvement in insect development and immunity. Understanding how the functions of similar MMPs vary with taxa from flies to beetles to butterflies may yield important information about how vertebrate MMPs evolved. In addition, the similarity between insect MMPs and vertebrate MMPs makes these a desirable model for testing hypotheses about MMP function. For example, if MMPs are involved in carbohydrate metabolism, at the cellular level, vertebrate functions may also be similar. Insects can also serve as useful models for innate immunity, since hemocytes are very similar to mammalian immune cells (Lemaitre and Hoffmann, 2007; Strand, 2008) and they both express MMPs (Johansson et al., 2005; Knorr et al., 2009). Finally, elucidating functions for MMPs in lower taxa may yield critical information about the evolution of the numerous MMPs found in vertebrates.
